# Significance of Anti-HLA Antibodies on Adult and Pediatric Heart Allograft Outcomes

**DOI:** 10.3389/fimmu.2017.00004

**Published:** 2017-01-27

**Authors:** Massimo Mangiola, Marilyn Marrari, Brian Feingold, Adriana Zeevi

**Affiliations:** ^1^Division of Transplant Pathology, Department of Pathology, University of Pittsburgh Medical Center, Pittsburgh, PA, USA; ^2^Pediatric Cardiology, The Children’s Hospital of Pittsburgh of UPMC, Pittsburgh, PA, USA

**Keywords:** AMR, heart transplantation, donor-specific antibodies, allograft vasculopathy, graft failure

## Abstract

As methods for human leukocyte antigens (HLA) antibody detection have evolved and newer solid phase assays are much more sensitive, the last 15 years has seen a renewed focus on the importance of HLA antibodies in solid organ transplant rejection. However, there is still much controversy regarding the clinical significance of antibody level as depicted by the mean fluorescence intensity of a patient’s neat serum. Emerging techniques, including those that identify antibody level and function, show promise for the detection of individuals at risk of allograft rejection, determination of the effectiveness of desensitization prior to transplant, and for monitoring treatment of rejection. Here, we review current publications regarding the relevance of donor-specific HLA antibodies (DSA) in adult and pediatric heart transplantation (HT) with graft survival, development of antibody-mediated rejection and cardiac allograft vasculopathy (CAV). The negative impact of DSA on patient and allograft survival is evident in adult and pediatric HT recipients. Many questions remain regarding the most appropriate frequency of assessment of pre- and posttransplant DSA as well as the phenotype of DSA memory vs. true *de novo* antibody using large multicenter adult and pediatric cohorts and state-of-the-art methodologies for DSA detection and characterization.

## Introduction

Heart transplantation (HT) has become an accepted therapy for adult and pediatric patients with end-stage heart failure. Despite improved immunosuppression regimens, rejection remains the most common cause of death in the first 5 years after HT. Both cellular and humoral immune-mediated processes that can damage the allograft are primarily directed against human leukocyte antigens (HLA). Antibodies against HLA can be found in patients prior to transplantation after exposure to foreign HLA through blood transfusion, pregnancy, previous transplant, and use of homograft tissue during surgery for some congenital heart defects. Ventricular assist devices (VAD) have also been implicated in the development of HLA antibodies, termed allosensitization. Exposure to donor HLA after HT may also induce *de novo* production of donor-specific HLA antibodies (DSA). The impact of circulating HLA antibodies on heart allografts has been the focus of many investigations and reviews. The introduction of solid phase assays (SPA) based on the luminex single antigen bead assay (SAB) has improved the sensitivity and specificity of HLA antibody detection; however, it also introduced new challenges for assay interpretation and determining its clinical relevance (1).

Identification of DSA enables the clinician to make informed decisions regarding acceptance of the organ and the choice of immunosuppression (2). Presence of DSA is not always considered a contraindication but rather a risk factor for organ transplantation success (3). Optimizing transplantation of allosensitized candidates is challenging and program specific. The main challenge with the new SPA technology is decision-making regarding donor organ acceptance based solely on antibody strength determined by mean fluorescence intensity (MFI) (2–5). The threshold for accepting a donor for a sensitized patient may vary depending on the patient’s clinical status, antibody level, and protocols available for antibody removal therapy. Considering the SPA modification to detect complement-fixing antibodies (C1q-SAB) has reduced the estimated incompatible donor pool in highly sensitized patients (6). Optimizing transplantation of allosensitized candidates using SAB and C1q-SAB methodology to prioritize the assignment of unacceptable antigens has allowed transplantation of highly allosensitized patients across the DSA barrier with survival rates comparable to DSA− heart transplant recipients (5).

Titration of sera prior to SAB testing has emerged as a more accurate way to assess the true level of DSA as compared to MFI value of undiluted sera (7). Furthermore, titration studies provide better estimates of responsiveness to antibody removal therapies (8).

Recognition that some preformed antibodies are against denatured HLA antigens with very little clinical relevance may also impact the search for an acceptable donor (4, 9). The assignment of unacceptable antigens has been greatly improved also by incorporating patterns of epitope reactivity and history of sensitizing events. Recognizing the limitations and advantages of current available methods for antibody determination, quantitation and function has facilitated the introduction of the virtual crossmatch (VXM) in thoracic transplantation. Previously, the need for prospective crossmatch (XM) in sensitized patients was associated with longer waitlist duration and increased mortality (10). Although VXM is widely used for organ allocation, its validity highly depends on how accurate and current is the information on patient sensitization events and comprehensive DNA-based HLA typing of prospective donors as antibodies can be made against every possible polymorphic HLA target antigen (2–5).

## Relevance of DSA on Outcomes

In this report, we focus on a short review of the current state-of-the-art regarding the role of DSA in adult and pediatric HT as determined by the following outcome measures: graft survival (GS), development of antibody-mediated rejection (AMR), and cardiac allograft vasculopathy (CAV) (Tables 1 and 2). Although we limit this review to the last 6 years, the retrospective nature of some studies may influence the relevance of DSA on clinical outcomes due to the use of less sensitive testing methods. Furthermore, we considered separately the role of DSA on adult and pediatric clinical outcomes to highlight potential similarities and differences in the two cohorts.

**Table 1:** **Cited publications from the last 6 years (2010–present) showing the impact of HLA antibody on heart transplantation in adult recipients**.

Reference	Number of patients (study period)	Method	DSA	GS	AMR	CAV	Comments
Gandhi et al. (19)	85 (August 2006–January 2010)	CDC-AHG PRA/XM, Flow XM, SAB	All CDC XM−; DSA+ (MFI >1,500), *n* = 11 (13%): Class I = 2, Class II = 6, Class I + II = 3		(*n* = 80 for biopsy) AMR: 7/11 DSA+		CMR ≥ 1R/1A: 9/11 DSA+ vs. 48/69 DSA−/weak; DSA MFI >1,500 associated with increased incidence of AMR and CMR

Smith et al. (14)	243 (October 1995–July 2004)	SAB (8.8 ± 2.5)	57 dnDSA Class II = 48 (42/48 DQ)	Poor GS *p* = 0.0001 (HR = 4.35)		29% 5 y; 55% 10 y	DnDSA risk for poor GS and CAV

Ho et al. (16)	950 (January 1995–December 2009)	CDC T and B, SAB (mean number of sera tested per patient = 24 ± 9)	221 dnDSA 1 y, 118 dnDSA >1 y, 460 no HLA-Ab	GS 52%, *p* < 0.005; GS 48%, *p* < 0.001; GS 70%	23		DSA and non-DSA increased in rejection

Loupy et al. (29)	196 (1985–2009)	SAB	20 very late rejection (VLR >7 y)			CAV grade VLR, 2.06 vs. 0.76 in control	VLR associated with severe CAV

Hodges et al. (15)	762 (November 2005–August 2011)	Luminex Screen, SAB	15 AMR (14/15 dnDSA)	1.8 y mean survival after AMR treatment	15		Late cardiac AMR with dnDSA

Zeevi et al. (20)	15 (8 pediatric, 7 adult)	SAB, SAB-C1q	35 DSA in 14 patients: Class I = 4, Class II = 2, Class I + II = 8		1st month post-Tx: 7/7 cAMR+ are DSA+/C1q+; 4 cAMR-free, DSA+/C1q− (*p* < 0.005)		Persistent C1q+ DSA post-Tx associated with early clinical AMR

Potena et al. (11)	173 (2000–2005)	CDC/PRA, Luminex Screen	Pre-Tx 32 Ab+ Class I = 28, Class II = 16, Class I + II = 12	Survival 65% for Ab+ 82% for Ab−	9/37 with biopsy were HLA-Ab+, pAMR >2		

Raess et al. (13)	272 (1989–2010)	CDC-PRA/XM, Luminex screen, SAB, SAB-C1q	DSA 26 (9.6%), Class I = 14, Class II = 5, Class I + II = 7, C1q+ DSA = 2	Overall survival: 80% (1 y), 68% (5 y) SAB Class I DSA+: 62% (1 y), 50% (5 y) SAB Class I DSA−: 87% (1 y), 73% (5 y)	Fatal pAMR = 6, all ≤1 month post-Tx	(*n* = 245) CAV-free survival: 96% (1 y), 86% (5 y)	ACR-free survival: 38% (1 y), 30% (5 y); pre-Tx HLA Ab status affected short-term survival but had no effect on long-term survival/rejection

Topilsky et al. (27)	51 (January 2004–December 2009)	SAB; Flow XM for 30 patients	All CDC-XM−; DSA+ 17 (33%): Class I = 4, Class II = 11, Class I + II = 2			36 (71%) with Grade 1 CAV	CAV analysis done for patients with only Class II DSA; pre-Tx Class II DSA may give higher risk of accelerated CAV: DSA+ 100% vs. DSA− 64.2% at 4 y

Tible et al. (22)	111 (October 2009–September 2010)	SAB, 150 paired DSA and EMB	47/150 DSA+, Class I = 40.4%, Class II = 40.4%, Class I + II = 19.2%		37		MI and CD68 associated with DSA+

Frank et al. (28)	109 (February 1996–June 2011)	SAB, 330 paired DSA and EMB	51/112, Class I = 5, Class II = 26, Class I + II = 20			24 (22%): 40% DSA+, 13% DSA−	33% with CAV pre-Tx DSA+; Class II DSA, IF C4d+, and MI high risk for failed allograft with CAV

Coutance et al. (24)	20 (November 2006–February 2013)	Luminex Screen, SAB	19/20 tested were dnDSA+	50% after 1 y	Late AMR (>1 y post-Tx)		Prognosis for late AMR poor despite aggressive therapy

O’Connor et al. (12)	12,858 (June 2004–March 2013); UNOS database	CDC-PRA, Flow-PRA	PRA ≥ 10%, Class I: CDC+ = 227, Flow+ = 2,243, Class II: CDC+ = 126, Flow+ = 2,218	PRA ≥ 10%: HR = 1.24 (95% CI 1.12–1.36)			Percent Ab+ patients increased from 2005 to 2011 as use of flow increased; pre-Tx PRA ≥ 10% by Flow associated with increased risk of graft loss

Svobodova et al. (21)	264 (April 2005–December 2012; mean follow-up 39 months, range 19–66)	CDC-PRA/XM; SAB, SAB-C1q	DSA = 28 (11%): Class I = 18, Class II = 3, Class I + II = 7, C1q+ DSA = 4	90% (1 y), 79% (5 y)	19 (7%)	31 (12%)	74 patients (28%) with 83 instances of ACR grade ≥ Banff 2; pre-Tx DSA and elevated peak CDC-PRA were strongest predictors of AMR

Frank et al. (23)	44 (2005–2011)	SAB-C1q paired with EMB C4d stain	C1q+ DSA in 82% with graft dysfunction	18/44 died or retransplanted	16/17 C4d+ IF had C1q+ DSA; 24 C1q+ DSA were C4d-IF		Better concordance of C4d+ IF with C1q DSA as compared to IgG DSA

Loupy et al. (25)	40, failing grafts	SAB			AMR = 19		

Clerkin et al. (26)	689 (January 2004–December 2013, follow-up through October 2015)	Luminex SAB and/or CDC screen	Overall: *n* = 29 (42.6%); early AMR: *n* = 22 (51.1%); late AMR: *n* = 7 (28.0%)	Decreased post-AMR survival in patients with late vs. early AMR: 80 vs. 93%, 1 y; 51 vs. 73%, 5 y (*p* < 0.05)	*n* = 68 (9.9%): 43 early (<1 y post-Tx), 25 late (>1 y post-Tx)	No difference in prevalence early AMR vs. late AMR (*p* = 0.51); accelerated *de novo* CAV in late AMR + graft dysfunction (50% at 1 y, HR = 5.42, *p* = 0.009)	Graft dysfunction increased in late AMR group (56.0 vs. 25.6%, *p* = 0.01)

*Ab, antibody; ACR, acute cellular rejection; AHG, anti-human globulin; AMR, antibody-mediated rejection; C1q, complement component 1q; C4d, complement component 4d; CAV, cardiac allograft vasculopathy; CDC, complement-dependent cytotoxicity; CMR, cell-mediated rejection; XM, crossmatch; DSA, donor-specific HLA antibodies; dnDSA, *de novo* donor-specific HLA antibody; EMB, endomyocardial biopsies; GS, graft survival; HR, hazard ratio; IF, immunofluorescence; MFI, mean fluorescence intensity; MI, microcirculation inflammation; pAMR, pathologic AMR; post-Tx, posttransplant; PRA, panel-reactive antibodies; pre-Tx, pretransplant; SAB, Luminex single antigen bead assay; VLR, very late rejection; y, year(s); HLA, human leukocyte antigens*.

**Table 2:** **Cited publications from the last 6 years (2010–present) showing the impact of HLA antibody on heart transplantation in pediatric recipients**.

Reference	Number of patients (study period)	Method	DSA	GS	AMR	CAV	Comments
Rossano et al. (31)	3,534 (October 1987–May 2004, follow-up through May 2008), UNOS database	CDC-PRA/XM most commonly used	PRA >10% = 387 (11%); 9% XM+	Median graft survival PRA >10% = 7.1 y PRA 1–10% = 9.6 y PRA 0% = 9.8 y			Decreased long-term GS in patients with PRA >10%

Irving et al. (38)	59, mean post-Tx follow-up 5.1 y (range 0.7–18.5 y)	Luminex screen/SAB	*N* = 4 (7%): 1 transient Class I, 3 persistent Class II	DSA+: 1/4 functioning, 2/4 retransplanted, and 1/4 died (7 y post-Tx)	DSA+: 2/4 (50%); non-DSA+: 1/15 (7%); no Ab: 5/40 (13%)	DSA+: 3/4 (75%); non-DSA+: 1/15 (7%); no Ab: 3/40 (7.5%)	Severe cellular rejection (≥3R) *n* = 3 (5.1%), all DSA−

Chin et al. (42)	18 (June 2007–February 2009)	CDC-XM, SAB, SAB-C1q, Flow CXM	SAB-IgG DSA: Pre-Tx 61.1%, Post-Tx 55.5%; SAB-C1q DSA: Pre-Tx 21.4%, post-Tx 35.7%	94% (1 y), 82% (2 y)	Within 1st month: *n* = 5 (27.7%), all post-Tx SAB-C1q+ DSA		SAB-C1q assay may better predict early AMR

Mahle et al. (32)	1,904 (January 1993–December 2008) Pediatric Heart Transplant Study Group	CDC-PRA most commonly used	PRA ≥ 10% = 397 (15.8%); PRA ≥ 50% = 189 (7.6%)	1 y patient survival: PRA ≥ 50%, 73 vs. 90% for PRA <10%		No CAV association with pre-Tx Ab	No association of PRA with time to 1st rejection or CAV

Ho et al. (16)	108 (January 2000–December 2009)	CDC-PRA, SAB	PRA >10% Class I = 9% Class II = 14%	87% GS in CDC− vs. 33% CDC+ after 7 y			Correlation between AMR and presence of CDC- or SPA-detected DSA

Scott et al. (34)	101 (2004–2008)	CDC-PRA, FLOW		PRA >25% decreased GS vs. patients with PRA <25%	*n* = 12: 33% with PRA >80% vs. 13% with PRA <80%		

Peng et al. (44)	60 (October 2005–January 2011)	FLOW-PRA, SAB, 183 paired DSA and C4d			6 (3/6 XM+)		Correlation between C4d+ in EMB and DSA >6,000 MFI

Daly et al. (58)	134 (January 1998–January 2011)	CDC-AHG PRA, Luminex SAB; XM+ patients received preoperative plasmapheresis + IVIG	12 XM+ (9%) T+/B+ = 8 T−/B+ = 2 T+/B not tested = 2	No significant difference in GS for XM+ (*n* = 3, 25%) vs. XM− (*n* = 12, 10%)	1 yr post-Tx: XM+ = 6 (50%), XM− = 2 (2%) (*p* < 0.001)		Serious infection higher in XM+ vs. XM− (50 vs. 16%, *p* = 0.005); shorter time to 1st infection in XM+ (*p* = 0.001)

Asante-Korang et al. (35)	70 (January 2005–July 2013)	Luminex PRA, SAB, Flow-XM; desensitization performed in patients with PRA >10%	PRA >10% = 14 (20%)	Overall patient survival: 92.9% in sensitized group vs. 80.4% in non-sensitized	Freedom from AMR or rejection grade ≥2R/3A: 71.4% in sensitized vs. 64% in non-sensitized	Freedom from CAV: 93% for sensitized vs. 91% in non-sensitized	12/14 high PRA patients had reduced Ab levels following desensitization; no significant differences in outcomes between desensitized patients and those with no Ab

Chen et al. (36)	25 (January 2008–June 2010)	PRA and SAB, 195 samples	12/25 dnDSA	No impact short-term survival			Majority of dnDSA within 1 y

Irving et al. (47)	108 (1996–2009)	SAB, 691 samples	43 DSA (58% persistent) Class I = 30% Class II = 47% Class I + II = 23%	9/14 with graft loss had persistent DSA		9/10 with CAV DSA+; 6/9 DSA persistent	Persistent DSA associated with poor outcome and CAV

Godown et al. (39)	121 (1987–2014), mean follow-up 4.1 y	Flow, Luminex, all were XM−	dnDSA: 40 (33%) Class I = 24% Class II = 50% Class I + II = 26%				Multiple factors influence DSA development; DSA seen more frequently in patients with prior sensitizing events

Ware et al. (43)	66 (January 2009–September 2013)	SAB	27 DSA+ (4 XM+)	No impact	DSA level associated with pAMR2, 3	No impact	Negative predictive value of DSA testing for absence of pAMR

Tran et al. (37)	105 (January 2002–December 2012, follow-up 0.13–10.8 y)	SAB (5 times first year and yearly after)	45 (43%) DSA Class I = 20% Class II = 62.2% Class I + II = 17.8%	5 y GS 72.4% DSA− vs. 21% DSA+		CAV 36% DSA+ vs. 13% DSA−	DSA+ had 2.5 times more rejection events per year compared to DSA−

Thrush et al. (40)	1,596 (January 2010–December 2014), Pediatric Heart Transplant Study database	Unknown		33 deaths (16%) post-AMR development; short-term patient/GS lower for patients with treated AMR (*p* = 0.004, *p* = 0.001, respectively); patient survival post-AMR diagnosis: 88% 1 y, 77% 3 y	179 (11%), freedom from AMR: 88% 1 y, 82% 3 y		AMR often concurrent with ACR

*Ab, antibody; ACR, acute cellular rejection; AHG, anti-human globulin; AMR, antibody-mediated rejection; C1q, complement component 1q; C4d, complement component 4d; cAMR, clinical AMR; CAV, cardiac allograft vasculopathy; CDC, complement-dependent cytotoxicity; XM, crossmatch; DSA, donor-specific HLA antibodies; dnDSA, *de novo* donor-specific HLA antibody; EMB, endomyocardial biopsies; GS, graft survival; HR, hazard ratio; IF, immunofluorescence; pAMR, pathologic AMR; post-Tx, posttransplant; PRA, panel-reactive antibodies; pre-Tx, pretransplant; SAB, Luminex single antigen bead assay; SPA, solid phase assays; y, year(s); MFI, mean fluorescence intensity; HLA, human leukocyte antigens*.

## Adult HT

### Graft Survival

The prevalence of allosensitization in heart transplant candidates increased with the introduction of SPA for screening for HLA antibodies (11) (Table 1). Nevertheless, the risk for poor GS has remained a significant finding even in the more sensitive SPA testing era (11, 12). The presence of non-cytotoxic HLA antibodies identified by SAB was associated with high risk of death, early graft failure, and late cellular- and antibody-mediated rejection; these findings underscore the need for using sensitive Luminex platform SPA to accurately determine the presence of circulating HLA antibodies (12). Detection of Class I DSA pretransplant was a predictor of short-term but not long-term survival as compared to non-DSA (13). In this study limiting the testing on pretransplantation, the authors could not identify the impact of persistent vs. transient DSA and of *de novo* DSA on clinical outcomes.

*De novo* antibody production and its role in cardiac allograft survival has been described in several studies (14–16). In a retrospective adult cohort, *de novo* DSA was associated with poor patient survival (HR = 3.198), while *de novo* and persistent DSA was worst (HR = 4.351) (14). Similarly, patients with persistent *de novo*, mostly Class II DQ-specific DSA, had worse survival (15). The 15-year survival was highest in patients who never developed DSA vs. those that developed DSA posttransplantation (70 vs. 47%), and patients with late *de novo* DSA appearing more than 1 year post transplantation had poorest survival (16). Thus, determining the presence of DSA pretransplant for risk assessment and monitoring for persistent and *de novo* DSA posttransplant provide the most comprehensive information for clinical management.

### Antibody-Mediated Rejection

The challenges of AMR diagnosis post HT have been addressed by many single-center studies and consensus conferences. In the current era, diagnosis of this clinically important entity has been improved by standardized classification of histologic and immunologic changes in endomyocardial biopsies (EMB) (17) and by advances in the detection of HLA antibodies. Although not required for diagnosis of pathologic AMR (pAMR), the detection of HLA antibodies pre- and posttransplantation has been helpful for risk stratification for the development of AMR and for guiding treatment strategies (18). Patients with positive VXM defined in the presence of DSA >1,500 MFI by SAB had a higher incidence of AMR and cell-mediated rejection. Similar outcomes were observed with positive flow crossmatch (FXM) suggesting that SAB MFI >1,500 can be used as surrogate for FXM (19). Increased risk for a positive complement-dependent cytotoxicity (CDC) XM and early AMR was observed in patients with persistent C1q+ DSA (20). However, patients who had DSA but lost the C1q reactivity posttransplant did not develop early AMR, and the strength of neat sera on SAB did not predict C1q reactivity. In contrast, high titer DSA (>1:16) has been associated with complement-fixing reactivity (7, 20) and has been used to determine unacceptable HLA antigens for sensitized candidates (5). DSA determination by SPA and elevated peak panel-reactive antibodies (PRA) were independent predictors of pAMR in an adult cohort of heart transplant recipients (21). In this study focusing on pretransplant samples, increasing numbers of DSAs and the mean cumulative MFI of DSAs were associated with risk of AMR, and the subset of C1q-reactive DSAs were less informative (21). Pathologic classification of AMR in 37 EMB correlated with circulating DSA and endothelial activation (22). The proportion of DSA+ EMB varied according to pAMR grade, and pAMR2 was associated with 100% DSA positivity (22). The clinical significance of DSA level as depicted only by MFI of neat serum is still controversial, and currently multiple approaches are proposed to capture the DSA level and function, including serum titration and complement-binding assays. A better concordance was observed between C1q+DSA and C4d immunofluorescence (IF)+ staining in EMB as compared with total IgG DSAs and C4d IF+ in EMB among 44 recipients (40 vs. 24%, *p* = 0.02) (23). A majority (82%) of patients with graft dysfunction had circulating C1q+ DSAs (23). However, not all patients with circulating C1q+DSA had C4d IF+ staining on EMB, suggesting that the presence of C1q+DSA may precede the development of pAMR or be due to the low sensitivity of C4d IF staining (23).

Prognosis after late AMR (defined as AMR >1 year posttransplant) was poor in 20 recipients despite aggressive treatment with immunosuppression, and fulminant CAV was a common condition (24). DSA was present in all tested patients (*n* = 19) with a median cumulative MFI at diagnosis >10,000; most of the patients had *de novo* DSA (24). Antibody-mediated injury and immune-mediated coronary arteriosclerosis were the causes of late graft failure in a recent study of 40 explanted heart allografts (25). AMR was observed in 47.5% failing heart allografts, including 40% of patients in whom unrecognized previous episodes of subclinical AMR occurred years before allograft loss. Among the 19 patients with AMR, 15 were tested for DSA, and 93% had circulating DSA at the time of allograft failure. The immunodominant DSA was Class II in 11/14 DSA+, and the median DSA MFI was 5,000 (25). In contrast, only 37% of patients without AMR features at the time of allograft failure had circulating DSA as compared to the AMR group (*p* < 0.001), and the median DSA MFI was 1,250 (25). In a retrospective cohort study spanning over 10 years, the timing of AMR (early vs. late) was associated with GS and CAV (26). Patients were tested at the time of biopsy for circulating DSA either by CDC (before 2010) or SAB (post 2010). Graft dysfunction was less frequent in early AMR, while late AMR with graft dysfunction showed rapid development of *de novo* CAV despite aggressive treatment and also increased risk of death (26).

### Cardiac Vasculopathy

Cardiac allograft vasculopathy continues to remain a limiting factor in long-term survival of heart transplant recipients, and there is increasing evidence of the negative impact of circulating DSA on the development and severity of CAV. Patients with DSA had significantly higher rates and a shorter mean time to CAV and increased severity of CAV as compared to patients without DSA (27, 28). Patients with very late rejection and circulating DSA with evidence of intravascular macrophages had an increased risk of severe CAV as compared to patients without DSA (29).

## Pediatric HT

### Graft Survival

Allosensitization and GS in pediatric HT recipients have been evaluated in large single- (30) and multicenter datasets (31, 32) (Table 2). Pediatric patients with PRA >10% had earlier-onset graft vasculopathy (30) and worse graft and patient survival than did patients with PRA <10% (31, 32). Elevated PRA was an independent risk factor for worse long-term GS (31). Furthermore, significant allosensitization (PRA >50%) at listing was associated with a more than twofold increased risk of death within the first transplant year (32). These large patient cohorts that were transplanted over a period of 18 years may have underestimated the rate of allosensitization because the methodology for PRA screening evolved from a less sensitive cell-based method to the more sensitive SPA. In addition, the SPA may have also increased the need for prospective XM due to an increased use of VXM (33).

In a more recent study patients with PRA >25% had significantly (*p* = 0.004) decreased survival compared to those with PRA <25% (34). In contrast, the outcome of allosensitized pediatric patients with PRA >10% who were desensitized was not different than non-sensitized recipients (35).

Assessments of GS in the presence of DSA show somewhat mixed findings, perhaps related to the duration of follow-up. Although short-term GS was not impacted by the presence of DSA in one pediatric study (36), the 5-year survival was significantly better in patients without DSA in another pediatric cohort (72 vs. 21%) (37). While uncommon, the presence of *de novo* DSA posttransplantation, especially toward Class II HLA, was associated with increased graft loss (38).

Multiple factors appear to play a role in development of *de novo* DSA in pediatric HT including prior sensitizing events, older age, African-American race, and donor death from gunshot wound (39). Knowledge of risk factors for the development of *de novo* DSA in pediatric recipients is likely to be important to guide the frequency of monitoring for HLA antibodies (39).

### Antibody-Mediated Rejection

Current understanding of AMR after HT is largely derived from adult studies. Using the Pediatric Heart Transplant Study database, the reported incidence of AMR was 11% (among 1,596 recipients), and patient and GS were lower for those with AMR (40). Risk factors associated with AMR included PRA >10% at HT, a positive CDC XM, and congenital heart disease, suggesting allosensitization related to the use of homografts (40).

The proportion of AMR-free patients was much higher among patients with only solid phase-detected DSA vs. those with CDC-detected DSA (41). Similarly, using the C1q assay, which detects only complement-fixing antibodies, the presence of C1q fixing DSA prior to or early after HT had a positive predictive value of 100%, while absence of C1q fixing DSA had a negative predictive value of 100% for AMR (42). In another analysis, the presence of circulating DSA had 93% sensitivity, 62% specificity, 24% positive predictive value, and 99% negative predictive value for biopsy diagnosis of AMR in pediatric recipients (43). In addition, higher levels of circulating DSA measured by MFI correlated with pAMR severity (43). The authors proposed that DSA monitoring provides a non-invasive tool to tailor the frequency of biopsy surveillance (43). Others have used an institution-specific MFI threshold value for DSA of >6,000 that strongly correlated with C4d deposition on EMB with high negative predictive value (97%) and specificity (95%) (44). Both studies emphasized the advantage of following DSA in asymptomatic pediatric patients, given the value of early detection of AMR (43, 44).

Similar to findings for renal transplantation (45), sensitized recipients with persistent posttransplant DSA with complement-fixing ability appear to be at high risk for AMR (20, 42, 46).

### Cardiac Vasculopathy

Overall, DSA+ patients (preformed or *de novo*) had significantly higher rates of CAV compared with DSA− patients. By 5 years, the rate of CAV-free survival was 25% for DSA− vs. 0% for DSA+ (37). Persistent DSA was associated with poor outcome and development of CAV (47).

## Treatment

Desensitization is aimed to increase the donor pool by either reducing or eliminating HLA allosensitization or by facilitating transplant by reducing the DSA burden. Desensitization treatment targets critical components of the humoral response to either achieve a negative crossmatch pretransplant or to reduce the impact of DSA in positive crossmatch transplants. At low titer, antibody reduction can be achieved with plasma exchange and IVIG. The use of B cell suppression agents (rituximab), plasma cell depletion agents (bortezomib), or inhibitors of complement activation (eculizumab) is usually limited to highly sensitized patients. The current literature in adults is not abundant, mostly observational, with small cohorts, short follow-up, and with inconsistent treatment methodologies (48–53). In 21 highly sensitized patients, the use of plasmapheresis (PP), IVIG, rituximab, and cyclophosphamide resulted in comparable long-term survival when compared to the low sensitized and unsensitized cohorts (53). A recent experience with bortezomib and PP showed that about 50% of the patients had a calculated PRA reduction and were transplanted with a negative crossmatch (48, 52). One year follow-up showed 100% survival and 74% freedom from rejection (48, 52). In a smaller cohort of patients transplanted across a positive crossmatch and treated with eculizumab and ATG, 1-year survival was 89%, and freedom from rejection was 75% (52). In patients treated for AMR, Class I HLA antibodies demonstrated a statistically significant response to bortezomib, whereas Class II responded poorly (51).

In pediatric HT, requiring a negative prospective crossmatch increases the waiting time and more importantly the waitlist mortality (10). Allosensitization is most significant among children with certain forms of congenital heart disease due to the use of homograft during prior surgeries. Also, blood transfusions and VAD use are common causes of allosensitization. Current literature for pediatric heart transplant desensitization is even more limited than in the adult cohort (54–58). Desensitization was carried out successfully with bortezomib and PP in a pediatric setting (54). Furthermore, in a single-center retrospective study in a large cohort of patients, all sensitized patients received PP or plasma exchange preoperatively. If the cytotoxic XM was positive, PP was continued. Patients with negative XM did not receive additional PP and IVIG posttransplant (58). Hemodynamically significant AMR occurred in 50% of patients transplanted across a positive XM vs. 2% of the XM-negative cohort (58). Additionally, incidence of serious infection was higher in patients transplanted across a positive crossmatch (58). Antibody depletion therapies were also used in management of AMR in pediatric patients. Decreased DSA MFI in 21 patients treated with PP correlated with good clinical outcome (55). In another small study, addition of bortezomib to PP and rituximab treatment resulted in a rapid decline in DSA and reversal of AMR without significant side effects (56).

## Summary

The negative impact of DSA on patient and allograft survival is evident in adult and pediatric HT recipients. Allosensitization depicted by PRA >10% using cell based (prior era) or SPA (current era) is associated with poor outcome in both cohorts. Furthermore, similar risk factors were identified in adults and pediatric recipients for the development of posttransplant DSA including sensitizing events pretransplant, ECMO, need for mechanical support, non-compliance, and African-American race. In adults, but not in pediatrics, female gender (prior pregnancies) was also associated with a higher risk for development of *de novo* DSA. In children, exposure to homografts as part of surgical repair for some forms of congenital heart disease increase their risk for allosensitization and AMR.

Many questions remain regarding the most appropriate frequency of assessment of pre- and posttransplant DSA as well as the phenotype of DSA memory vs. true *de novo* antibody using large multicenter adult and pediatric cohorts and state-of-the-art methodologies for DSA detection and characterization. The observation that early vs. late AMR in HT may have different prognosis and responses to treatment emphasizes the need to assess the risk of sensitization pretransplantation and to follow by routine monitoring of DSA posttransplant.

The ongoing multicenter clinical collaborative studies supported by National Institute of Health in adult and pediatric HT will hopefully provide answers to many remaining questions regarding the impact of preformed and *de novo* DSA on clinical outcomes and the efficacy of various modalities for desensitization and treatment of AMR.

## Author Contributions

This is an invited review on impact of HLA donor-specific antibody in cardiac allograft outcome. All authors contributed equally to literature review, summary, and manuscript preparation.

## Conflict of Interest Statement

The authors declare that the research was conducted in the absence of any commercial or financial relationships that could be construed as a potential conflict of interest.
